# Primary prevention in general practice – views of German general practitioners: a mixed-methods study

**DOI:** 10.1186/1471-2296-15-103

**Published:** 2014-05-25

**Authors:** Christine Holmberg, Giselle Sarganas, Nadine Mittring, Vittoria Braun, Lorena Dini, Christoph Heintze, Nina Rieckmann, Rebecca Muckelbauer, Jacqueline Müller-Nordhorn

**Affiliations:** 1 Berlin School of Public Health, Charité Universitätsmedizin Berlin, Seestr. 73, 13347 Berlin, Germany; 2 Institute for Social Medicine, Epidemiology, and Health Economics, Charité Universitätsmedizin Berlin, Luisenstr. 57, Berlin 10117, Germany; 3 Institute of General Practice, Charité Universitätsmedizin Berlin, Chariteplatz 1, Berlin 10117, Germany

**Keywords:** General practice, Primary prevention, Physician-patient relationship

## Abstract

**Background:**

Policy efforts focus on a reorientation of health care systems towards primary prevention. To guide such efforts, we analyzed the role of primary prevention in general practice and general practitioners’ (GPs) attitudes toward primary prevention.

**Methods:**

Mixed-method study including a cross-sectional survey of all community-based GPs and focus groups in a sample of GPs who collaborated with the Institute of General Practice in Berlin, Germany in 2011. Of 1168 GPs 474 returned the mail survey. Fifteen GPs participated in focus group discussions. Survey and interview guidelines were developed and tested to assess and discuss beliefs, attitudes, and practices regarding primary prevention.

**Results:**

Most respondents considered primary prevention within their realm of responsibility (70%). Primary prevention, especially physical activity, healthy eating, and smoking cessation, was part of the GPs’ health care recommendations if they thought it was indicated. Still a quarter of survey respondents discussed reduction of alcohol consumption with their patients infrequently even when they thought it was indicated. Similarly 18% claimed that they discuss smoking cessation only sometimes. The focus groups revealed that GPs were concerned about the detrimental effects an uninvited health behavior suggestion could have on patients and were hesitant to take on the role of “health policing”. GPs saw primary prevention as the responsibility of multiple actors in a network of societal and municipal institutions.

**Conclusions:**

The mixed-method study showed that primary prevention approaches such as lifestyle counseling is not well established in primary care. GPs used a selective approach to offer preventive advice based upon indication. GPs had a strong sense that a universal prevention approach carried the potential to destroy a good patient-physician relationship. Other approaches to public health may be warranted such as a multisectoral approach to population health. This type of restructuring of the health care sector may benefit patients who are unable to afford specific prevention programmes and who have competing demands that hinder their ability to focus on behavior change.

## Background

According to the World Health Organization (WHO) 2008, approximately 80% of heart disease, stroke, and type-2-diabetes could be prevented globally with an elimination of risk factors such as smoking, physical inactivity, and unhealthy diet [1]. Thus, the WHO and other health services bodies have argued for a focus on behavior change and health promotion in health care services [2-7]. The debate on how such a change may take place and how GPs can be included in the effort to reorient the health care system towards primary prevention is ongoing [8-16]. Specific barriers to an inclusion of primary prevention in primary care have been identified such as an already overburdened health service and the perceived limited effect a GP has on a patient’s behavior [8,10,13,17-20]. In addition, structural and systemic difficulties in reorienting health care systems play a role. For example, in Germany a reorientation of the health care system requires federal legislation. To date, legislation regarding primary prevention is mostly aimed at individuals and companies. The German reimbursement structure for health care does not consider primary prevention targeting patients’ lifestyle as a reimbursable task of GPs, except as a small part of the nationwide health check-up screening programme [21].

Interestingly, surveys find that the overwhelming majority of GPs in Germany and across Europe support and believe in primary prevention but many of them do not include primary prevention efforts, especially prevention related to lifestyle change, in their practice [12,13,22-24]. This gap between beliefs and practice raises questions regarding the role of primary prevention in GPs’ offices. Thus far, only a few studies have looked at the practice of primary prevention in GPs’ offices [25-28]. The purpose of this study was to understand current primary prevention practices in general care and the attitudes and beliefs that GPs in Germany hold about primary prevention.

## Methods

### Design and procedures

In order to achieve a complete picture of the practice of primary prevention in general practice as well as to learn about GPs’ attitudes, beliefs, and behaviors with regards to primary prevention we conducted a mixed-method study including a cross-sectional survey and focus groups. The survey was necessary for a quantitative assessment of the prevalence and practice of primary prevention in primary care. The aim of the focus groups was to explore GPs’ practices and beliefs regarding primary prevention from their point of view and in an exchange of opinions. Recruitment took place between November 2010 and February 2011.

For the survey component of the study we contacted all general practitioners who owned a practice in the Greater Berlin area through the Berlin Chamber of Physicians. Similarly, the focus group members were part of this general population. However, to facilitate recruitment, we contacted GPs who work in close collaboration with the Institute of General Practice at the Charité - Universitätsmedizin Berlin to invite them to the focus group discussions.

### Data collection and analysis

#### Survey

The Berlin Chamber of Physicians contacted all GPs with a private practice in Berlin. The GPs received an information letter, a questionnaire, and a response envelope by mail (N = 1168). To increase participation the Dillmann method was used [29]. Thus, non-responders were contacted twice after the initial invitation to study participation. The first reminder consisted of a postcard. Those, who did not respond to the postcard, received the questionnaire once again four weeks after the initial mailing.

The aim of the survey was to assess current primary care activities (as indicated by patient referrals and lifestyle counseling), and to capture GPs’ perceptions about responsibilities in primary prevention (as indicated by assumptions and beliefs regarding primary prevention courses for patients). The survey was developed based on existing questionnaires from the Bertelsmann Stiftung [12] and was modified to fit the research question of this study. The questionnaire was pre-tested for comprehensibility among GPs practicing outside Berlin by the means of cognitive interviews (n = 5), and piloted among 20 other GPs who completed and commented on the questionnaire. Basic characteristics of our source population, all GPs residing in Greater Berlin, were provided by the Berlin Chamber of Physicians. Data analysis was performed using SPSS Statistics 19.

#### Focus groups

The Institute of General Practice at the Charité - Universitätsmedizin Berlin contacted 130 general practitioners teaching surgeries in Berlin by email and invited them to participate in the focus groups. Four focus groups were organized so that all GPs who responded to the call could participate in the discussions.

The aim of the focus group was to understand 1), how GPs currently integrate primary prevention into their practice and 2), the attitudes and beliefs they hold towards primary prevention in general practice. The focus groups were led by a trained moderator. After an introduction into the topic of research, participants were asked to discuss their daily work experiences with primary prevention, the role they wanted primary prevention to play in the GP practice, knowledge about existing prevention programs that were partially reimbursed by health insurance companies, barriers that existed in conducting prevention, and suggestions to improve the delivery of primary prevention in the work of GPs. Even though priorities of themes shifted between focus groups, all four focus group discussions addressed similar aspects of primary prevention in general practice. Therefore, we decided to end recruitment after the fourth focus group discussion.

After each focus group discussion the moderator wrote a summary in which she reflected on the interpersonal aspects of the discussion. She described her role in the group as well as group dynamics she experienced. The discussions were digitally recorded, transcribed verbatim, and entered into the software program MAXQDA for qualitative analysis. Two members of the research team coded the materials (C.H. and N.M.). Materials were coded according to the topics of the interview guideline. Then codes and categories were developed that emerged from the data; differences in coding were resolved in discussions. Categories and themes were refined in comparative analysis [30]. Data was compared on several levels: First the entire focus group discussions were compared to each other according to themes that emerged from the discussions as well as the dynamics the moderator experienced or observed during the discussions. Secondly, the segments of individual codes were compared and contrasted to each other in order to understand the discussants’ current practices of primary prevention in general practice and to analyse their beliefs and views regarding primary prevention in general practice. The analysis was conducted jointly by C.H. and N.M. to ensure intersubjectivity. To further ensure the adequacy of the analysis, anonymized qualitative data and results were regularly discussed in a qualitative research group that was not associated with the research project. These procedures ensured the appropriateness and reliability of the analysis. The quotes presented in this paper are representative of how the emerging themes were discussed in the focus groups.

The study was approved by the Charité Universitätsmedizin Berlin Ethics Committee (EA1/249/10 and EA2/135/10). Since the survey component of the study was anonymous, no written consent was necessary. Focus group participants gave written informed consent prior to the study.

## Results

### Sample

A total of 474 GPs responded to the survey (response rate = 41%). Sample characteristics of respondents as well as focus group participants are displayed in Table 1.

**Table 1:** Characteristics of survey respondents and focus group participants

	**Survey respondents n = 474**	**Focus group participants n = 15**	**Source population (Berlin chamber of physicians) n = 1168**
**Age**	Frequency (%)	Frequency (%)	Frequency (%)
< 35	0	0	1 (0,1)
35 – 49	182 (38)	6 (40)	319 (27)
50 – 64	235 (50)	6 (40)	662 (57)
≥ 65	50 (11)	0	186 (16)
Missing	7 (2)	3 (20)	-
**Sex**			
Male	152 (32)	3 (20)	494 (42)
Female	313 (66)	9 (60)	674 (58)
Missing	9 (2)	3 (20)	-
**Practice type**			
Individual	266 (56)	5 (33)	941 (81)
Group	185 (39)	3 (20)	216 (18)
Ambulatory healthcare center	9 (2)	3 (20)	11 (1)
Missing	14 (3)	4 (27)	-
**Patient structure**^**1**^	Mean (SD^2^)	Mean (SD^2^)	
Statutory health insurance patients	87 (18)	92 (7)	-
Patients with private health insurance	13 (18)	8 (7)	-

^1^The percentage of patients in each general practice according to insurance status.

^2^Standard deviation.

### Survey

**Figure 1:**
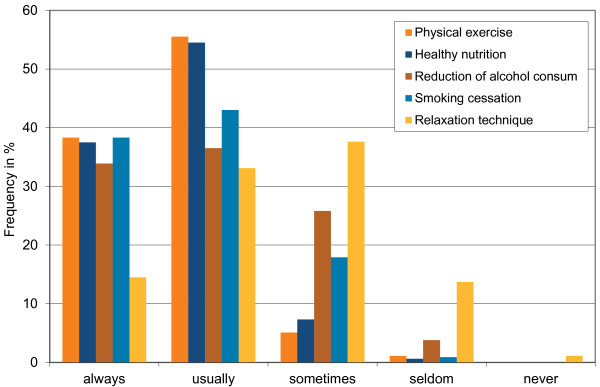
Frequency of how often GPs addressed primary prevention behaviours when indicated.

**Figure 2:**
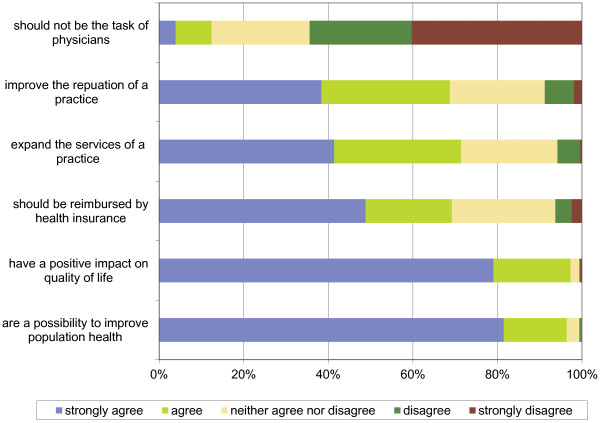
Beliefs about primary prevention.

Primary prevention, especially physical activity, healthy eating, and smoking cessation, was part of the GPs’ health care recommendations if they thought it was indicated (Figure 1). Interestingly, a quarter of the sample discussed reduction of alcohol consumption infrequently even when they thought it was indicated. Similarly, 18% of respondents discussed smoking cessation only sometimes even when they thought it was indicated.Most participants considered promoting primary prevention as part of their role as GPs (70%) but also thought that each individual (74%), schools and day-care centres (57%), health insurance companies (42%), and public health services (36%) were responsible for primary prevention. Almost all GPs (96%) believed that primary prevention offers an avenue to promote population health and could have a positive influence on quality of life (Figure 2).

### Focus groups

#### Current situation and barriers to addressing prevention in the GP practice

##### Prevention practices

Each of the fifteen participants in the focus group discussions considered primary prevention important. Changes in lifestyle such as smoking cessation, healthy eating, and physical activity were specifically mentioned as fields of intervention. Although vaccination was named as a primary prevention tool by participants, it was not discussed further. Vaccination was considered part of their tasks and did not pose any difficulties for their clinical practice. In contrast, lifestyle interventions as a potential element of clinical practice were discussed extensively. Discussants regularly distinguished between primary and secondary prevention when case examples were presented in the group. For example, when one participant described a case from their practice as a prevention activity, others discussed the type of prevention the example illustrated. It became apparent that most of the examples discussants provided belonged to the category of secondary prevention.

Person 1: What is quite difficult [to achieve in general practice] is to influence people’s lifestyle. This lies in the nature of general practice where people come with an illness. They do not come to be counselled about lifestyle, [they come to be treated for an illness].

*Person 2: Especially since in general practice a discussion on lifestyle only begins when a health care problem exists, regardless if it is high blood pressure or diabetes. This poses the question if this is indeed primary prevention?!* (focus group 2)

Most GPs in the focus groups agreed that the only time they might discuss lifestyle changes without a particular indication was with those patients who presented for the health check-up.

Person 2: I think, the only time I do talk about primary prevention in the practice is at the health check-up to which we invite patients. (focus group 1)

At the same time, some agreed that patients coming to health check-up did not need lifestyle counseling.

Person 2: Those that come to the health check-up are those that really do not need counselling on lifestyle issues.

Person 3: Exactly.

*Person 4: Usually those are the ones that are healthy and who are very aware of their lifestyle and health issues.* (focus group 2)

### Barriers to primary prevention in general practice

The fact that primary prevention overall was seldom practiced in general practice was mainly associated with the structure of general practice in Germany. Four structural issues were highlighted: 1), The organization in the office of a general practitioner, 2), the reimbursement scheme for general practitioners, 3), the role of a general practitioner and their view of patients, and 4), the socioeconomic circumstances of patients.

1) The organization of the office

General practitioners overall had high patient volume which led to long waiting times for patients. Therefore persons who were not ill were unlikely to come to the office.

*Person 2: Healthy people will do everything but certainly not sit in a full waiting room.* (focus group 2)

Indeed, some of the discussants had changed the structure of their practice to allow for a larger amount of primary prevention activities by means of the healthy check-up that was reimbursable for patients 35 and older. One of them had implemented a system that all patients aged 35 and older were reminded of the check-up; another GP had reserved one afternoon solely to conduct such health check-ups. This also allowed for patients to return for several visits during the time they tried to change behavior.

*Person 3: I decided that I want to focus my practice on primary prevention. However, to do so, I changed the structure of my practice. Because it does need time. So you need to organize the practice in such a way that you are able to discuss primary prevention options.* (focus group 4)

Person 1: We do a lot of check-ups. We actively talk to our patients to encourage them to participate. The entire practice team.

Person 2: Yes, we also have a system to record when the check-up was done and when it should be done the next time.

Person 3: So what do you then with the check-ups? Do you tell them to be physically active for example?

*Person 1 and 2: Of course!* (focus group 4)

2) Reimbursement scheme

The way the practice was organized was inherently connected to the existing reimbursement scheme. Primary prevention is not part of this schema, except for the above-mentioned health check-up.

Lack of time was considered as another factor that hindered primary prevention efforts in general practice. All discussants agreed that successful primary prevention required time to talk about it with patients. Similarly, if someone wanted to change their behavior, this needed follow-up appointments in order to ensure long-term behavior change through continued motivational support. Such close monitoring was neither part of the present reimbursement scheme nor was it feasible due to the high patient volumes in the offices.

Person 4: We usually include lifestyle counseling in our regular routine. But when it is actually happening when someone for example, wants to stop smoking but needs our help, it becomes difficult. What do we do with such a person? Shall I just give him a prescription, period? Usually such an approach does not help the patient to quit smoking. So, we have to invest time and see the patient several times and talk with him. But how shall we take the time for these conversations?

*Person 1: Yes, primary prevention is not part of our job description or our reimbursement schema. We deal with disease. So primary prevention really does not play a role in our practice, except that we mention it to people.* (focus group 4)

3) Perceptions regarding their role as GP and their view of patients

All discussants mentioned lifestyle changes to their patients when they thought it was appropriate. Appropriateness was assessed based on the reason of the visit and the patient.

*Person 1: I will talk about smoking if someone comes with bronchitis or a cold. Then I ask if they smoke and that they need to quit smoking in the future. Otherwise it will not go away. Especially if someone comes with a cold and if the patient can then hear a noise from their own breathing and one listens to the lungs and is able to say, listen your bronchial airways are damaged. Such a symptom makes it more palpable to the patient if I talk about smoking to them.* (focus group 1)

For most of the GPs, the decision to discuss lifestyle changes was based on the specific issue with which the patient presented. For example, if a patient presented with a disease that was affected by unhealthy lifestyle habits (for example, bronchitis and cigarette smoking), GPs would take the opportunity of the situation and address lifestyle changes. Similarly, it seemed easier for GPs to discuss lifestyle changes when a patient had symptoms that would be alleviated once lifestyle changes were adopted. However, some of the discussants thought it was possible to introduce lifestyle issues more generally but they questioned the effectiveness of such an approach.

*Person 4: Of course I can talk about weight reduction to a patient who comes because of a sickness. The question though is how effective is it?!* (focus group 1)

Most participants argued against a routine discussion of behavior changes in general practice for two reasons: a), patients needed a desire to modify their behavior, and b), physicians were critical of taking on a role of “health policing.”

a), Participants believed that in order for behavior change to be successful it had to be motivated from within oneself and needed to be relevant to the patient’s life. Such a motivation could be encouraged by the GP if the lifestyle change was related to the reason for the health care visit. For instance, most GPs agreed that they would not discuss smoking cessation with a patient who presented with back pain. However, they would certainly talk about physical activity and weight reduction in such a situation.

*Person 1: For example, if someone comes because of a cold and I notice that this person smokes. I can talk about smoking [because it may influence the duration of the cold]. But of course, you cannot talk to a person about safer sex who comes because of a cold.* (focus group 2)

They also thought that the will of the patient to change was important. This was another reason why many of the discussants did not consider a standard discussion of behavior change useful in general practice.

*Person 3: Yes, I do believe that the will to change has to come from the person herself. It has to be their project.* (focus group 1)

*Person 3: I think, the fear of growing old and becoming frail and in need of help is something that leads people to change their behavior or to participate in courses on behavior change. Others will not do it.* (focus group 2)

b) Some of the discussants were against a standardized approach to lifestyle intervention in general practice based on the perception they had of general practice. They were critical of a normative understanding of how people should live their life based on health. Some discussants questioned the role of a GP as someone overseeing how adults should live their lives.

*Person 3: I am not sure if we should put health first place. Of course, physicians always do that. But there are people who don’t. There are people who want to smoke and they know this may mean that they do not live as long. And I think it should not be the task of primary prevention to change people’s behavior if they do not want to do so. These are adults and they have the right to choose.* (focus group 1)

Person 2: Sometimes primary prevention is very normative about how one should live. And it actually can be quite nice to not live according to health principles. Still, we should address it when appropriate but not in a standardized format. We don’t have to save people. I don’t want a patient to leave my office with a feeling of being converted.

Person 1: Indeed, I find it quite interesting to ask about the focus of primary prevention in the legal realm. They target smoking cessation, physical activity, losing weight. However, being happy and quality of life are not part of this approach. (…)

*Person 1: Yes, and it is important that we as physicians also accept the ways people live and still take good care of them. There should not be a value associated with these behaviors.* (focus group 1)

4) Competing demands: The socioeconomic circumstances of patients

Socioeconomic circumstances were seen as a barrier to target behavior change. GPs’ patients discussed precarious financial and social conditions. This led to what participants identified as “competing demands.” Such patients faced many challenges in their lives such that changing an unhealthy lifestyle was a low priority and one that was difficult to argue for from the GP’s perspective.

*Person 4: The social situation in which the patients are, no money available, and also to quit smoking is so much more difficult, if you are out of work and sitting at home, your electricity has been cut. This burden is too much for a physician’s office it is not the realm we can address. To help with all of these social aspects is not possible for us. And then other problems are simply more important than smoking.* (focus group 1)

### Suggestions for ways to integrate primary prevention into general practice

During the focus groups GPs shared their visions and suggestions for successful primary prevention involving general practice. These are presented in Table 2 along with relevant quotes.

**Table 2:** Suggestions for improving primary prevention efforts in general practice: quotations of the most salient focus group results

**Suggestions for improvement by GPs**	
- Creation of a larger primary prevention network	*“I mean, there have to be structural changes, so that the primary prevention is defined as a medical job (…). And I could also imagine a collaboration between the offers of the health insurance companies and local communities. (…) And locally. Not anywhere in the middle of the city in a big institution, where all people have to collect, but it has to happen where people work, where they live. In the neighbourhood.”* (Person 1, Focus Group 4)
- Increase of primary prevention efforts in a network of community planning, infrastructure, and schooling	*“The statutory health insurance had a program in schools, where they sent physicians to class as well as to teachers to discuss [primary prevention]. So I went into a primary school class and talked about nutrition. (..) After the class, I had a counseling session with each student one-on-one and we talked about eating disorders. And one of them knew exactly that he was eating because of stress and what he should be doing instead. And I think that is the way to go. We need to go to schools (Person 4: Yes, we should go to schools). There we can still influence kids. The experience at the time was great. The students were very open and interested.”* (Person 2, Focus Group 3)
- Reimbursement structure that allows for follow-up meetings and counselling sessions on behavior change	

In the discussions, primary prevention was seen as a task that extended beyond the responsibility of medical practice. Improving patients’ health was seen as a joint endeavor of doctors, communities, schools, day care centers and individuals.

GPs suggested the development of a network of stakeholders to foster successful primary prevention including collaborations between GPs, paediatricians, communities, and health insurance companies to organize programs that fit the needs of each individual community.

As mentioned before, most patients served by GPs were ill and often presented with multiple ailments. For that reason, GPs felt that primary prevention as behavior change needed to be introduced at an earlier stage in life. The ideal target groups for primary prevention were children and young adults. These age groups were rarely in contact with a GP. Children were usually seen by paediatricians and young adults did not frequently access the services of health care providers. Thus public infrastructure and school programs were seen as crucial settings for primary prevention.

Finally, GPs suggested a restructuring of the GP reimbursement schemes in ways that allowed a successful and effective prevention focus in practice and recognition of prevention as a medical task.

Most of the GPs agreed that their training qualified them to provide information on the physical effects of unhealthy behaviors. Explaining the physical consequences of unhealthy behavior in various settings, including schools and daycare centers in addition to their practices, was seen as the way they could offer support in prevention networks.

## Discussion

The discussion of health behaviors and techniques of primary prevention was part of the GPs’ routine practice. Primary prevention was seen as an effective tool to improve population health and quality of life and GPs usually included health care recommendations such as physical activity, healthy eating, and to a lesser degree smoking cessation into their care when they thought it was indicated. Other studies in Germany and the Netherlands also have found that GPs include primary prevention into their care when they perceive it as indicated or when patients ask for them [31,32]. The high level of addressing physical activity as a preventive health measure is comparable to findings from Brazil [33]. Similarly, other studies suggest that the lifestyle intervention that is most commonly addressed by general practitioners is physical activity. Advice on alcohol consumption and smoking cessation is not as common [34-36].

Our findings from the focus groups may help explain why smoking cessation and alcohol consumption may be handled differently in clinical practice. In the focus groups that we conducted the routine introduction of primary prevention was not seen as feasible or even desirable for some. The interviewed GPs perceived general discussions of behavior change without an indication as inappropriate in most instances. Similarly, some felt uneasy in a role of enforcing behavior change on their patients. Others have found that general practitioners need an occasion to discuss behavior change and Abholz [37] has identified such an approach a “specific general practitioner prevention.” He used this term to classify a particular narrative approach GPs may use to deal with patients’ complaints in which GPs take up what patients tell them during the health care visit. Streich and Stock [22] have shown the ways such narrative approaches may include aspects of prevention similar to the ones that discussants of the focus groups mentioned. However, we argue that due to the broader debate on the reorientation of health care delivery towards prevention, using the term prevention to describe GPs’ practices may be misleading. GPs clearly stated that their focus was not primary prevention but secondary prevention at best. Such findings have been shown by other survey studies in a German GP population [38]. The focus group participants in our study considered carefully when, and whether or not, to include primary preventive efforts in relation to the GP-specific patient-doctor relationship and their self-perceived role as a GP. These considerations of general practitioners should be taken seriously not only in terms of their own perceived role as GP but with respect to their view of their patients as wilful and independent beings as well.

Other studies have shown that GPs use the reason for the health care visit as an anchor for behavior change discussions [19,26,39]. A qualitative study on patients’ experiences with lifestyle counseling found that patients wanted to be recognized in their concrete life situation and needed a good personal doctor-patient relationship for a discussion to be helpful [40]. This speaks to the individually tailored approach GPs in this study utilized to discuss lifestyle changes.

However, no study has yet discussed the fact that standardized approaches to primary prevention in general practice do not merely add an additional task for the GP but instead change the role of the GP toward their patients. The relationship becomes a moral one in which one, the GP, explains to another how to live one’s life. Healthism has been analyzed and critiqued in general [41]. It may be pertinent to add studies that examine how the patient-doctor relationship is affected when primary prevention that exclusively focuses on behaviour change is introduced.

While we contacted all GPs in Berlin for the survey only 41 percent responded. Compared to the source population, our sample was younger and we had a larger proportion of female physicians. It is very likely that respondents were highly interested in primary prevention resulting in a responder bias such that non-responders are less likely give primary prevention advice to their patients. Other studies have found female physicians in Germany to be more active in providing lifestyle counseling to patients, especially on dietary habits, which reiterates that our sample was more prone towards primary prevention than the general population of GPs in Germany [38]. Response rates to physician surveys are generally low, especially when no compensation is given. Our response rate is comparable or better to other studies conducted in Germany or the Netherlands [12,31]. Compared to the GP population of a rural area of Germany, the Berlin source population has a similar age distribution and fairly similar distribution of individual and group practices [42,43]. Similarly, a study that investigated GPs’ willingness to delegate primary prevention tasks to physician assistants found that GPs consider lifestyle counseling as part of their task. This suggests that attitudes and beliefs about primary prevention may not differ between physicians working in rural or urban areas [44]. Focus group participants were a highly select group that committed several hours of their time to the study. We must assume that they were highly invested in primary prevention or that they held strong views regarding primary prevention. This needs to be kept in minds when considering the recommendations made by focus group members such as the introduction of prevention networks. Other GPs may not be motivated to join such networks. Similarly, focus group participants were invested in the education of future general practitioners, which may mean that they are more interested in new developments in practicing medicine compared to other physicians. This again suggests that a different sample may not agree with their suggested improvements to bring primary prevention into practice. Focus group participants were successfully recruited from different suburban and urban areas with practices that included patients with diverse socioeconomic and ethnic backgrounds. Thus their actual experiences with primary prevention are influenced by a diverse set of factors. Finally, GPs’ own health behaviors were not assessed. These have an influence on what they present to patients. This could be an explanation of why many of the respondents did not address lifestyle changes even if they thought they were indicated. However, since we assume that our responders are in fact more interested in primary prevention, this is unlikely. Presented findings need to be supplemented by studies on patients’ views, especially regarding a standardized discussion of health behaviors. A strong patient-physician relationship is the foundation of a physicians’ influence on patients’ behaviors. It thereby needs to be carefully evaluated if it may be detrimental to initiate a conversation about behavior change with all patients. Similarly, GPs’ fears need to be addressed if primary prevention efforts shall be strengthened.

## Conclusion

Our research aimed to address the practice of primary prevention in GP practices. However, it became evident from the focus groups that primary prevention as universal counseling of all patients irrespective of risk status is not established in practice and is not desired by the interviewed GPs. GPs use a selective approach to offer preventive advice based upon indication. Indeed, GPs had a strong sense that a universal prevention approach potentially destroys a good patient-physician relationship. Johansson et al. [35] have suggested that the continuity in the patient-general practitioner relationship is favorable to introduce lifestyle counseling, but may have a detrimental effect on behaviors that are perceived as more sensitive such as smoking and drinking. Our findings support such an assumption. Since the difficulty of discussing smoking and alcohol consumption seems to be a phenomenon across many countries[33-36], our findings may also have broader applications. Considering the scarce evidence as to whether or not a reorientation of health care services towards prevention reduces the burden of disease, [45-47] other approaches may be warranted such as a multisectoral approach to population health [48-51]. This type of restructuring may benefit patients who are unable to afford specific prevention programmes and who have competing demands that hinder their ability to focus on behavior change.

## Competing interest

The authors declare that they have no competing interests.

## Authors’ contributions

CH worked on the conception and design of the study and analysed and interpreted the data and synthesized results of the mixed-method study. CH wrote the article. GS worked on the conception and design of the study, and analysed and interpreted the survey data. GS contributed significantly to the paper. NM coded and analysed the focus group data and revised the article critically for important intellectual content. VB worked on the conception and design of the study. LD collaborated on the survey development and revised the article critically for important intellectual content. CH collaborated on the conduct of the focus groups and their analysis and provided critical input for the analysis and revised the manuscript for important intellectual content. NR provided critical input for the analysis and revised the manuscript for important intellectual content. RM revised the manuscript for important intellectual content. JMN designed the study and was involved in all research steps, including analysis and interpretation of data. JMN revised the article critically for important intellectual content. All authors read and approved the final manuscript.

## Pre-publication history

The pre-publication history for this paper can be accessed here:

http://www.biomedcentral.com/1471-2296/15/103/prepub
